# Health Literacy, Psychological Distress and Fertility Attitudes Among Postpartum Women: A Mediation Analysis

**DOI:** 10.3390/healthcare14162444

**Published:** 2026-08-07

**Authors:** Ante Buljubašić, Vesna Antičević, Rahela Orlandini, Mirela Sušac, Iris Jerončić Tomić

**Affiliations:** 1 Faculty of Health Sciences, University of Split, 21000 Split, Croatia; vanticev@fzz.unist.hr (V.A.); rorlandi@fzz.unist.hr (R.O.); 2 Faculty of Health Studies, University of Mostar, 88000 Mostar, Bosnia and Herzegovina; mirela.susac@fzs.sum.ba; 3 School of Medicine, University of Split, 21000 Split, Croatia; iris.jeroncic@mefst.hr

**Keywords:** fertility attitudes, health literacy, postpartum women, anxiety, depression, psychological distress, mediation analysis, reproductive health

## Abstract

**Background/Objectives:** Declining fertility rates pose a major demographic and public health challenge across Europe, including Croatia. Although health literacy and psychological well-being may influence reproductive decision-making, their relationships with fertility attitudes remain insufficiently understood. This study examined the associations between health literacy, psychological distress, and fertility attitudes among postpartum women and investigated whether anxiety and depressive symptoms mediate the relationship between health literacy and fertility attitudes. **Methods:** A cross-sectional study included 1656 postpartum women from seven coastal counties in Croatia. Participants completed validated measures of health literacy (NVS-HR), anxiety (STAI), depressive symptoms (BDI-M), and breastfeeding attitudes (IIFAS). Correlation analyses, multivariable logistic regression, and mediation analyses were performed. **Results:** Higher health literacy was associated with more positive fertility attitudes (r = 0.18, *p* < 0.001), whereas anxiety and depressive symptoms were negatively associated with fertility attitudes (all *p* < 0.001). Health literacy was also negatively correlated with anxiety and depressive symptoms. Multivariable logistic regression demonstrated that higher health literacy (OR = 1.24, 95% CI 1.12–1.35), older age (OR = 1.08, 95% CI 1.05–1.12), and more positive breastfeeding attitudes (OR = 1.04, 95% CI 1.01–1.09) were independently associated with preferences corresponding to above-replacement fertility, whereas higher levels of depressive symptoms were independently associated with lower odds of this outcome (OR = 0.78, 95% CI 0.66–0.90; all *p* < 0.05). Mediation analysis demonstrated a significant direct association between health literacy and fertility attitudes (β = 0.020, *p* = 0.004), whereas no significant indirect effects through anxiety or depressive symptoms were observed. **Conclusions:** Health literacy was independently associated with fertility attitudes and with preferences corresponding to above-replacement fertility, while psychological distress did not mediate this relationship. These findings suggest that health literacy may serve as an important cognitive resource for reproductive decision-making and a potential target for future reproductive health promotion among postpartum women.

## 1. Introduction

Declining fertility rates represent one of the most significant public health and demographic challenges facing contemporary societies [1]. Over the past six decades, the total fertility rate (TFR) across Europe has declined from approximately 2.6 children per woman in the early 1960s to around 1.4–1.5 in recent years, remaining well below the replacement level of 2.1 children per woman [2,3,4]. Croatia belongs to the group of European countries with very low fertility rates, with the total fertility rate in recent years remaining around 1.4 children per woman, substantially below the level needed to maintain a stable population [5,6]. In Croatia, the TFR declined from 2.24 in 1960 to approximately 1.46 in 2023–2024, falling below the replacement level as early as 1968 [4,6]. Although Croatia’s TFR does not meet the stricter demographic threshold used to define “lowest-low fertility” (TFR ≤ 1.3) [7], it remains persistently and substantially below the replacement level. This demographic situation is comparable to that observed in several Southern and Eastern European countries, including Italy, Spain, Greece, and Poland, which continue to experience persistent below-replacement fertility and similar demographic challenges [2,3,4,5,6]. Several of these countries currently report TFRs below 1.3, reflecting an even lower level of fertility than that observed in Croatia [8].

Contemporary fertility research indicates that reproductive behaviour is not determined solely by biological factors but rather reflects a complex interplay of sociodemographic, economic, cultural, health-related, and psychological influences [9,10,11]. Particular attention has been directed in recent years toward fertility attitudes, which are considered important determinants of reproductive intentions and future reproductive behaviour in women [12]. Negative attitudes toward parenthood, feelings of uncertainty, and reduced perceived control over one’s life circumstances may significantly influence decisions to postpone or limit childbearing [13]. Consistent with the Theory of Planned Behavior, attitudes toward parenthood and fertility represent an important psychological component of reproductive decision-making because they contribute to the formation of reproductive intentions and subsequent reproductive behavior [12,13,14].

Beyond sociodemographic and economic factors, increasing attention has been directed toward the role of health-related factors in shaping reproductive attitudes and behavior [15,16,17]. Health literacy is an important public health concept that encompasses an individual’s capacity to access, understand, evaluate, and apply health information needed to make appropriate health-related decisions [15,16,17,18]. Higher levels of health literacy are associated with healthier behaviours, greater use of preventive health services, and a stronger ability to make informed decisions regarding reproductive health [16,19,20]. Women with lower health literacy may have difficulty understanding information related to reproductive health, pregnancy, and parenthood, which may increase uncertainty and negatively influence attitudes toward future childbearing [19,20,21]. Previous studies have consistently demonstrated that adequate health literacy is associated with improved reproductive health knowledge, greater utilisation of preventive reproductive healthcare, healthier pregnancy-related behaviours, and more informed reproductive decision-making. Empirical evidence directly linking health literacy with fertility attitudes, however, remains limited and has been concentrated primarily among university student populations. A study among Polish female students found that reproductive health knowledge was incomplete and inconsistent, with particularly poor awareness of lifestyle-related factors affecting fertility—knowledge gaps that may directly shape attitudes toward future childbearing [22]. Similarly, a study among Danish university college students demonstrated that limited fertility awareness was associated with unrealistic expectations regarding postponed parenthood, suggesting that health-literacy-related knowledge gaps can distort reproductive attitudes and intentions [23]. However, these studies were conducted almost exclusively among young, nulliparous student populations rather than postpartum women, whose reproductive attitudes are shaped by lived childbirth experience rather than anticipatory planning. To date, no study has examined the relationship between health literacy and fertility attitudes specifically among postpartum women, nor has psychological distress been investigated as a potential mediating mechanism in this relationship [21,22,23,24]. Consequently, the relationship between health literacy and fertility attitudes remains insufficiently explored, particularly among postpartum women [23,24].

Psychological status may also play a significant role in shaping reproductive attitudes [12,14,25]. The postpartum period is associated with an increased risk of anxiety and depression, which can negatively affect perceptions of parenthood, quality of life, and women’s reproductive plans [26,27,28]. Previous research has shown that psychological distress can influence reproductive health decision-making, although the mechanisms through which this occurs remain incompletely understood [11,29]. Psychological distress may represent one pathway through which health-related factors influence women’s reproductive attitudes, whereby lower health literacy may be associated with higher levels of anxiety and depression and, consequently, with less favourable fertility attitudes. From a theoretical perspective, health literacy may influence fertility attitudes indirectly by shaping women’s ability to understand and evaluate reproductive health information. Women with limited health literacy may experience greater uncertainty, lower perceived control, and increased emotional burden when making reproductive decisions. These psychological responses may subsequently influence attitudes toward parenthood and future childbearing. Accordingly, anxiety and depressive symptoms were hypothesised as potential mediating mechanisms through which health literacy may be associated with fertility attitudes [17,19,29].

Collectively, previous research suggests that fertility attitudes arise from the interaction of demographic, socioeconomic, psychological, and health-related influences. However, these determinants have generally been investigated separately rather than within an integrated conceptual framework. Most available studies have focused on fertility intentions, reproductive outcomes, or isolated psychosocial determinants, whereas comparatively little attention has been paid to the combined role of health literacy, psychological distress, and other modifiable health-related factors in shaping fertility attitudes. Consequently, the mechanisms linking cognitive resources, emotional well-being, and reproductive attitudes remain insufficiently understood, particularly during the postpartum period.

In Croatia, most fertility studies have focused primarily on demographic and economic aspects of fertility, whereas fewer have simultaneously addressed the psychological and health-related factors associated with fertility attitudes [5,30,31,32]. Available evidence suggests that studies examining health literacy, psychological distress, and fertility attitudes within a unified mediation model among postpartum women remain scarce. There is also a particular lack of research in the coastal region of Croatia, which, due to a prolonged pattern of very low fertility and specific demographic characteristics, is considered one of the most demographically vulnerable regions in the country.

Although previous research has separately linked health literacy and psychological distress with reproductive outcomes, there is a notable lack of studies examining their joint relationship in the context of fertility attitudes [20,23,29]. In particular, limited evidence exists regarding whether psychological distress may act as a mediating mechanism through which health literacy is associated with fertility attitudes among postpartum women [5,6,30]. Understanding this pathway is important because it may identify modifiable targets for interventions aimed at improving reproductive health literacy and psychological well-being during the postpartum period. This conceptual framework is consistent with contemporary models of health literacy, which view health literacy as a determinant of cognitive and psychological processes underlying health-related decision-making, as well as with theoretical models of reproductive decision-making suggesting that psychological well-being influences the translation of knowledge and perceptions into reproductive attitudes and intentions. However, because empirical evidence integrating these constructs remains limited, particularly among postpartum women, the proposed mediation model should be regarded as hypothesis-driven and exploratory.

Based on this rationale and previous evidence, the following research aims were formulated:

1. To assess levels of health-related variables (health literacy, fertility attitudes, and breastfeeding attitudes) and psychological characteristics among postpartum women.
2. To examine the role of demographic characteristics, health literacy, anxiety, depressive symptoms, and breastfeeding attitudes as factors independently associated with fertility preferences corresponding to above-replacement fertility (three or more desired children) using logistic regression analysis.
3. To investigate whether psychological distress mediates the relationship between health literacy and fertility attitudes among postpartum women.

## 2. Materials and Methods

### 2.1. Participants

This cross-sectional study included postpartum women hospitalized after childbirth in healthcare institutions across the coastal counties of Croatia (Zadarska, Šibensko-Kninska, Splitsko-Dalmatinska, Dubrovačko-Neretvanska, Primorsko-Goranska, Istarska, and Ličko-Senjska). Eligible participants were women aged 18 years or older who were able to understand the study information and independently complete the questionnaire in Croatian. Women with severe psychiatric or medical conditions that could impair comprehension or questionnaire completion, as well as those with incomplete questionnaire responses, were excluded.

The required sample size was determined a priori based on recommendations for multivariate and mediation analyses and was calculated using Cochran’s formula for estimating proportions with a finite population correction [33]. The sample was obtained using convenience sampling with prior stratification proportional to the number of deliveries in each of the observed counties [34]. A total of 1656 postpartum women participated in the study. The mean age of participants was 30.6 years (SD = 5.2). Detailed sociodemographic characteristics are presented in Table 1.

### 2.2. Procedure

Participants were recruited consecutively between the second and fourth postpartum day. Data were collected using standardized paper-based questionnaires. Prior to data collection, designated hospital employees were instructed by the principal investigators regarding participant recruitment and study procedures. Each eligible participant received a questionnaire package in an envelope containing the study information sheet, informed consent form, and questionnaires.

Participants completed the questionnaires independently in their hospital rooms and returned them immediately after completion in sealed envelopes to the designated hospital employee. This procedure ensured the anonymity and confidentiality of all responses. Participation was voluntary and anonymous, and written informed consent was obtained from all participants prior to enrolment.

### 2.3. Ethical Considerations

The study was conducted in accordance with the STROBE guidelines [35] and the Declaration of Helsinki [36]. Ethical approval was obtained from the Ethics Committees of all participating institutions: the Ethics Committee of Pula General Hospital (Approval No. 2168/01-59-79-112-25-13, 31 January 2025); the Ethics Committee of the University Hospital Centre Rijeka (Approval No. 2170-29-02/1-21-2, 31 May 2021); the Ethics Committee of Zadar General Hospital (Approval No. 02-3673/21-4/21, 11 June 2021); the Ethics Committee of Šibenik-Knin County General Hospital (Approval No. 01-80/1-21, 28 April 2021); the Ethics Committee of the University Hospital Centre Split (Approval No. 2181-147/01/06/M.S.-21-02, 28 May 2021); and the Ethics Committee of Dubrovnik General Hospital (Approval No. 01-49/4.4-21, 7 June 2021).

No personally identifiable information was collected, and all data were analysed at the aggregate level for research purposes only. Data protection was ensured in accordance with the General Data Protection Regulation (GDPR; Regulation (EU) 2016/679) and applicable national legislation [37,38]. Access to the dataset was restricted to the research team, and all data were securely stored and processed to maintain confidentiality.

### 2.4. Measures

#### 2.4.1. Sociodemographic Questionnaire

A structured sociodemographic questionnaire developed for this study was used. It collected data on age, place of residence, marital status, educational level, employment and economic status, number of children, and selected reproductive characteristics. These variables were used to describe the study sample and to examine their associations with fertility attitudes.

#### 2.4.2. Fertility Attitudes Questionnaire

Fertility attitudes were assessed using a structured questionnaire adapted from the instrument developed for the nationally representative Croatian Fertility Attitudes Survey conducted by Akrap in 2021–2022 [39]. The original questionnaire was developed for a nationally representative survey of reproductive-age adults in Croatia and was designed to comprehensively assess multiple dimensions of fertility attitudes within the Croatian demographic context. Specifically, it examined attitudes toward childbearing, parenthood, and population policy among adults of reproductive age.

For the present study, the questionnaire was contextually adapted for postpartum women while preserving the conceptual framework of the original instrument. Item wording was modified only where necessary to ensure relevance for women who had recently given birth without changing the theoretical meaning of the statements. Accordingly, the adaptation represented a contextual and linguistic modification rather than the development of a new measurement instrument.

The questionnaire comprised five sections (A–E), including sociodemographic characteristics, fertility intentions, and attitudes toward fertility, childbearing, and population policy. The attitudinal component consisted of 61 statements rated on a five-point Likert scale ranging from 1 (strongly disagree) to 5 (strongly agree). These items were organised into four theoretically defined domains: perceived value of children and parenthood (10 items), factors influencing childbearing decisions (14 items), reasons for having or postponing additional children (24 items), and attitudes toward population policy measures (13 items). Negatively worded items were reverse coded before analysis so that higher scores consistently reflected more positive fertility attitudes.

An overall fertility attitude score was calculated as the mean of all attitudinal items, in line with the multidimensional conceptualisation of fertility attitudes in the original national survey. Although the questionnaire encompasses four theoretically defined domains, these domains were conceptualised as complementary components of a broader latent construct reflecting overall fertility attitudes rather than as independent subscales. Consequently, consistent with the conceptual framework of the original national survey, an overall composite score was calculated and used in the primary analyses. The satisfactory internal consistency observed in the present study further supported this approach.

For statistical analyses, the continuous composite score was used in descriptive, correlational, and mediation analyses. In addition, one item assessing the desired total number of children (“How many children do you want to have?”) was used to classify participants into below-replacement (one or two children) and above-replacement (three or more children) fertility preference groups for logistic regression analysis. This categorisation was based on the demographic concept of replacement fertility and was intended to identify factors associated with above-replacement fertility preferences from a public health perspective.

Accordingly, two complementary outcome measures derived from the Fertility Attitudes Questionnaire were used in this study. The continuous composite fertility attitude score was analysed to examine overall variation in fertility attitudes and their associations with health literacy and psychological distress. In contrast, the binary fertility preference outcome was specifically created to identify factors associated with preferences corresponding to above-replacement fertility (three or more desired children), a distinction considered particularly relevant from both demographic and public health perspectives.

Because the questionnaire was adapted from an existing nationally applied Croatian instrument rather than developed de novo, the present study focused primarily on evaluating its internal consistency within the study population rather than repeating a full psychometric validation. The overall attitudinal scale demonstrated good internal consistency (Cronbach’s α = 0.83), supporting the reliability of the composite fertility attitude score in postpartum women.

##### Binary Fertility Preference Outcome

For the logistic regression analysis, fertility preference was operationalised as a binary outcome variable based on participants’ responses to the question “How many children do you want to have?”. Women reporting a desired family size of one or two children were classified as having below-replacement fertility preference, whereas those reporting a desire for three or more children were classified as having above-replacement fertility preference. This categorisation was based on the demographic concept of replacement fertility (2.1 children per woman) and was used to identify factors associated with fertility preferences corresponding to above-replacement fertility [3,4].

#### 2.4.3. Health Literacy Questionnaire (NVS-HR)

Health literacy was assessed using the Croatian version of the Newest Vital Sign (NVS-HR), a validated measure of functional health literacy. The instrument includes six items based on the interpretation of a nutrition label. Scores range from 0 to 6, with higher scores indicating higher health literacy. Internal consistency (Cronbach’s α) in the original Croatian validation study was 0.76 [40,41]. Cronbach’s α in the present study was 0.78.

#### 2.4.4. Anxiety Questionnaire (STAI)

Anxiety was measured using the State-Trait Anxiety Inventory (STAI), which comprises 40 items assessing state (STAI-S) and trait (STAI-T) anxiety. Each subscale yields scores ranging from 20 to 80, with higher scores indicating greater anxiety [42,43]. Cronbach’s α in the present study was 0.91.

#### 2.4.5. Depression Questionnaire (BDI-M)

Depressive symptoms were assessed using the modified Beck Depression Inventory (BDI-M), consisting of 21 items rated on a four-point scale. For the purpose of analysis, item responses were averaged, yielding a mean score ranging from 1 to 4, with higher values indicating more severe depressive symptoms [44,45]. Cronbach’s α in the present study was 0.89.

#### 2.4.6. Breastfeeding Attitudes Questionnaire (IIFAS)

Breastfeeding attitudes were measured using the Iowa Infant Feeding Attitude Scale (IIFAS), a 17-item instrument rated on a five-point Likert scale. Total scores range from 17 to 85, with higher scores reflecting more positive attitudes toward breastfeeding [46,47]. Cronbach’s α in the present study was 0.84.

### 2.5. Statistical Analysis

Statistical analyses were performed using IBM SPSS Statistics for Windows, Version 22.0 (IBM Corp., Armonk, NY, USA) [48,49].

Descriptive statistics were used to summarize participants’ characteristics and study variables. Continuous variables are presented as means and standard deviations (SDs) or medians with interquartile ranges (IQRs), depending on data distribution, whereas categorical variables are presented as frequencies and percentages. Normality of continuous variables was assessed using the Kolmogorov–Smirnov test and visual inspection of histograms.

Associations between continuous variables were examined using Pearson’s or Spearman’s correlation coefficients, depending on data distribution.

Binary logistic regression analysis was performed to identify factors independently associated with preferences corresponding to above-replacement fertility. The dependent variable was fertility preference, classified as below-replacement fertility preference or above-replacement fertility preference. Predictor variables included age, educational level, economic status, place of residence, health literacy (NVS-HR), state anxiety (STAI-S), trait anxiety (STAI-T), depressive symptoms (BDI-M), and breastfeeding attitudes (IIFAS). Age, educational level, economic status, and place of residence were entered into the model as ordinal variables, whereas NVS-HR, STAI-S, STAI-T, BDI-M, and IIFAS scores were entered as continuous variables. Results are presented as adjusted odds ratios (ORs) with corresponding 95% confidence intervals (95% CIs). Model calibration was assessed using the Hosmer–Lemeshow goodness-of-fit test, whereas overall model performance was evaluated using classification accuracy and the area under the receiver operating characteristic (ROC) curve (AUC).

Mediation analysis was performed to examine whether psychological distress mediated the association between health literacy and fertility attitudes. Health literacy (NVS-HR) was entered as the independent variable, fertility attitudes as the dependent variable, and state anxiety (STAI-S), trait anxiety (STAI-T), and depressive symptoms (BDI-M) as potential mediators. Indirect effects were estimated using bootstrap resampling with 5000 samples and bias-corrected 95% confidence intervals (95% CIs). Mediation was considered statistically significant when the bootstrap confidence interval did not include zero.

All statistical tests were two-tailed, and statistical significance was defined as *p* < 0.05.

## 3. Results

### 3.1. Descriptive Statistics of Health-Related and Psychosocial Variables

Descriptive analysis of the measurement instruments indicated that participants had a generally satisfactory level of health literacy, low to moderate levels of psychological distress, and predominantly positive attitudes toward breastfeeding. Results for the applied measurement instruments are presented in Table 2.

The mean NVS-HR score indicated an adequate level of health literacy among participants. STAI scores reflected moderate anxiety, whereas BDI-M scores indicated a low average level of depressive symptomatology in this postpartum sample. Breastfeeding attitudes, assessed using the IIFAS, were predominantly positive.

Additional analysis of depressive symptomatology showed that the largest proportion of participants fell within the minimal depression category (77.3%), whereas mild, moderate, and severe depression were considerably less common (Table 3).

Overall, the descriptive findings indicate a relatively favourable psychological and health profile among the study participants, characterised by adequate health literacy, low levels of depressive symptoms, and predominantly positive breastfeeding attitudes.

### 3.2. Factors Associated with Fertility Attitudes Among Postpartum Women: Demographic, Health-Related, and Psychological Factors

In accordance with the second research aim, which examined the role of demographic characteristics, health literacy, anxiety, depressive symptoms, and breastfeeding attitudes as factors associated with preferences corresponding to above-replacement fertility, associations between health literacy, psychological distress, and fertility attitudes were first examined using correlation analysis (Table 4).

A statistically significant positive association was found between health literacy and fertility attitudes, indicating that participants with higher health literacy generally reported more favourable fertility attitudes. Negative associations were also observed between anxiety, depression, and fertility attitudes, suggesting that greater psychological distress was associated with less favourable reproductive attitudes.

In addition, health literacy was negatively associated with psychological distress. Participants with lower health literacy had higher scores on anxiety and depression scales, suggesting that limited health literacy was associated with less favourable psychological status.

The correlation analysis further demonstrated that higher levels of anxiety and depression were associated with less favourable fertility attitudes, whereas higher health literacy was associated with more positive reproductive attitudes. Among the psychological variables, depressive symptomatology showed the strongest negative association with fertility attitudes.

These findings provided the basis for the subsequent multivariable logistic regression and mediation analyses.

Multivariable logistic regression analysis was subsequently performed to identify factors independently associated with preferences corresponding to above-replacement fertility. The binary outcome variable distinguished women reporting a desired family size of one or two children from those expressing a preference for three or more children.

The overall logistic regression model was statistically significant (χ^2^ = 47.56; df = 9; *p* < 0.001). Although the explained variance was modest (Nagelkerke R^2^ = 0.039), the model demonstrated acceptable calibration according to the Hosmer–Lemeshow goodness-of-fit test (χ^2^ = 9.68; *p* = 0.65) and was limited to discriminative ability (AUC = 0.63; 95% CI: 0.57–0.68). Overall, the model correctly classified 66.5% of participants, with a sensitivity of 42.7% and a specificity of 81.6%.

Analysis of individual variables demonstrated that older age, higher health literacy, more positive breastfeeding attitudes, and lower levels of depressive symptoms were independently associated with preferences corresponding to above-replacement fertility. In contrast, educational level, economic status, place of residence, and both state and trait anxiety were not independently associated with the outcome after adjustment for the other variables included in the model (Table 5).

Overall, the findings indicate that health literacy remained an independent factor associated with preferences corresponding to above-replacement fertility even after adjustment for demographic and psychological characteristics. Furthermore, lower levels of depressive symptoms and more positive breastfeeding attitudes were also independently associated with the outcome.

### 3.3. The Mediating Role of Psychological Distress in the Association Between Health Literacy and Fertility Attitudes

Mediation analysis was conducted to examine the potential mediating role of psychological distress in the relationship between health literacy and fertility attitudes. Health literacy (NVS-HR) was entered as the independent variable, fertility attitudes as the dependent variable, and anxiety (STAI-S and STAI-T) together with depressive symptoms (BDI-M) as potential mediators.

The results showed that health literacy had a statistically significant direct association with fertility attitudes, with higher health literacy being associated with more positive fertility attitudes. Health literacy was also negatively associated with anxiety and depressive symptoms, indicating that participants with higher health literacy generally reported lower levels of psychological distress.

However, bootstrap analysis of the indirect effects did not confirm a statistically significant mediating role of psychological distress. The bootstrap confidence intervals for all indirect effects included zero, indicating that neither anxiety nor depressive symptoms significantly mediated the association between health literacy and fertility attitudes (Table 6).

The mediation analysis therefore did not support the hypothesis that psychological distress represents an intermediary mechanism linking health literacy with fertility attitudes in this sample of postpartum women. Although anxiety and depressive symptoms were individually associated with fertility attitudes, their indirect effects did not reach statistical significance.

These findings suggest that health literacy appears to be associated with fertility attitudes primarily through a direct pathway rather than indirectly through psychological distress. This finding highlights the potential importance of health literacy as an independent factor associated with fertility attitudes among women during the postpartum period.

## 4. Discussion

### 4.1. Main Findings of the Study

This study examined health literacy, psychological distress, breastfeeding attitudes, and fertility attitudes among postpartum women in Croatia. Participants generally reported adequate health literacy, low levels of depressive symptoms, moderate anxiety, and positive breastfeeding attitudes. Three findings are particularly noteworthy. First, health literacy was positively associated with fertility attitudes and remained independently associated with preferences corresponding to above-replacement fertility after adjustment for demographic and psychological characteristics. Second, depressive symptoms and breastfeeding attitudes were independently associated with preferences corresponding to above-replacement fertility, whereas anxiety was significant only at the correlational level. Third, psychological distress did not mediate the relationship between health literacy and fertility attitudes.

The logistic regression model explained only a small proportion of the variance in preferences corresponding to above-replacement fertility (Nagelkerke R^2^ = 0.039). This finding is not unexpected because fertility attitudes and reproductive preferences are complex phenomena influenced by numerous factors beyond those included in the present study [50]. For example, relationship quality, partner support, socioeconomic expectations, reproductive history, family planning intentions, housing conditions, personal values, cultural and religious beliefs, parity, pregnancy planning, previous infertility, and childbirth experiences may all contribute to reproductive decision-making. As these variables were not collected, they could not be included in the multivariable model. Consequently, the modest explanatory power of the model should not be interpreted as indicating that the identified factors are unimportant, but rather as reflecting the multifactorial nature of fertility attitudes and reproductive preferences. Future studies incorporating these variables may provide a more comprehensive understanding of fertility attitudes among postpartum women.

It should also be noted that the logistic regression model examined factors associated with preferences corresponding to above-replacement fertility, whereas the continuous fertility attitude score was analysed separately in the correlation and mediation analyses. Together, these findings suggest that both cognitive and psychological factors are associated with fertility attitudes, while health literacy appears to represent a distinct cognitive resource whose association with fertility attitudes is not explained by psychological distress.

### 4.2. Sociodemographic, Health-Related and Psychological Factors Associated with Fertility Attitudes

The multivariable logistic regression analysis demonstrated that preferences corresponding to above-replacement fertility among postpartum women were associated with a combination of sociodemographic, health-related, and psychological factors. Among the sociodemographic characteristics included in the model, only age remained independently associated with the outcome after adjustment for the remaining variables. Specifically, older women were more likely to report preferences corresponding to above-replacement fertility, whereas educational level, economic status, and place of residence were not independently associated with the outcome.

These findings suggest that although sociodemographic characteristics are traditionally recognised as important determinants of reproductive behaviour, their independent contribution may be attenuated after accounting for health-related and psychological characteristics. Nevertheless, their inclusion in the multivariable model was essential for controlling potential confounding and obtaining more robust estimates of the independent associations between the examined variables and preferences corresponding to above-replacement fertility [51,52,53].

The finding that age remained independently associated with the outcome is consistent with previous studies reporting that reproductive intentions and desired family size change across the reproductive life course, reflecting differences in life experience, relationship stability, and perceived readiness for parenthood [51,54]. Conversely, the absence of independent associations for educational level, economic status, and place of residence suggests that these characteristics may influence fertility attitudes indirectly through health-related, psychosocial, or contextual pathways rather than exerting direct effects [52,53]. This finding further illustrates the multifactorial nature of fertility attitudes and emphasises the importance of simultaneously considering sociodemographic, health-related, and psychological characteristics when investigating reproductive decision-making [53,54,55].

Health literacy emerged as one of the key factors independently associated with preferences corresponding to above-replacement fertility. Women with higher health literacy reported more favourable reproductive attitudes regardless of their psychological status. This finding is consistent with contemporary public health models that conceptualise health literacy as an essential resource for informed health-related decision-making [16,54]. Women with higher health literacy may be better able to understand reproductive health information, evaluate potential risks and benefits, and effectively navigate healthcare services [56]. These competencies may enhance confidence in reproductive decision-making and contribute to more favourable attitudes toward parenthood [57]. Conversely, limited health literacy may increase uncertainty and reduce perceived control over reproductive choices [58]. Although previous research has primarily focused on demographic, economic, and social determinants of fertility, the present findings suggest that health literacy should also be considered an important factor associated with reproductive attitudes [59].

Depressive symptoms were independently associated with less favourable reproductive attitudes. This finding is consistent with previous studies demonstrating that psychological distress may influence perceptions of parenthood, future planning, and reproductive intentions [60,61,62]. Anxiety was also negatively associated with fertility attitudes in the correlation analysis; however, this association disappeared after adjustment for other variables. This suggests that depressive symptoms may be more relevant than anxiety when considering fertility attitudes during the postpartum period [63,64,65,66].

Positive breastfeeding attitudes also emerged as an independent factor associated with preferences corresponding to above-replacement fertility. Women expressing more favourable attitudes toward breastfeeding may also hold more positive perceptions of motherhood and parenting, consistent with previous findings [67,68,69,70,71]. This observation highlights the potential importance of breastfeeding attitudes within the broader context of reproductive decision-making.

Beyond health literacy, psychological factors, and breastfeeding attitudes, women’s reproductive attitudes may also be influenced by their experiences of pregnancy, childbirth, and perinatal healthcare. Positive childbirth experiences, including effective labour pain management and high maternal satisfaction with obstetric care, may promote more favourable perceptions of childbirth and influence future reproductive attitudes. Consistent with this concept, Gunduz et al. reported that walking epidural analgesia using either ropivacaine or bupivacaine provided effective analgesia with high maternal satisfaction and no adverse maternal or neonatal outcomes, highlighting the importance of high-quality obstetric care in shaping positive reproductive experiences [72]. Although these aspects were beyond the scope of the present study, they represent important contextual factors that should be considered in future research examining fertility attitudes and reproductive decision-making.

The findings may also be interpreted within the framework of the Theory of Planned Behavior. According to this model, attitudes and perceived behavioural control contribute to behavioural intentions [12]. Health literacy may strengthen perceived behavioural control by improving women’s ability to obtain, understand, evaluate, and apply reproductive health information [73,74,75]. This mechanism may partly explain the observed association between health literacy and more favourable fertility attitudes.

### 4.3. Psychological Distress as a Mediator Between Health Literacy and Fertility Attitudes

One of the objectives of this study was to examine whether psychological distress mediates the relationship between health literacy and fertility attitudes. Although health literacy was associated with lower levels of anxiety and depressive symptoms, mediation analysis did not support a statistically significant indirect effect [16,76]. These findings suggest that psychological distress is unlikely to represent the primary mechanism linking health literacy with fertility attitudes in this sample. Instead, health literacy demonstrated a direct association with fertility attitudes.

Women with higher health literacy may develop more favourable reproductive attitudes through greater knowledge, confidence, and perceived control rather than through reductions in psychological distress [77]. Nevertheless, the absence of a significant mediation effect does not diminish the importance of mental health. Both anxiety and depressive symptoms were associated with fertility attitudes at the bivariate level, while depressive symptoms remained independently associated with the outcome in the multivariable model. Psychological well-being therefore remains an important component of reproductive health, although it did not explain the relationship between health literacy and fertility attitudes in the present study [78,79].

Because data were collected during the immediate postpartum period, limited variability in psychological distress may also have contributed to the absence of statistically significant mediation effects. Furthermore, because the present study was based on cross-sectional data, the mediation analyses should be interpreted as statistical associations consistent with the proposed conceptual model rather than as evidence of temporal or causal mediation. Consequently, although the mediation model was theoretically justified, it should be regarded as hypothesis-generating rather than confirmatory. Future prospective longitudinal studies are needed to establish the temporal sequence among health literacy, psychological distress, and fertility attitudes and to determine whether psychological distress genuinely mediates this relationship over time.

### 4.4. Scientific and Public Health Contribution of the Study

This study contributes to the literature by integrating health literacy, psychological distress (anxiety and depressive symptoms), breastfeeding attitudes, and fertility attitudes within a single analytical framework among postpartum women. To our knowledge, this is among the first studies in Croatia and one of the few internationally to examine whether psychological distress mediates the relationship between health literacy and fertility attitudes during the postpartum period.

The findings demonstrate that health literacy is independently associated with fertility attitudes, whereas psychological distress does not significantly mediate this relationship. These findings suggest that health literacy may represent a distinct cognitive resource relevant to reproductive decision-making. Furthermore, the study provides evidence from a large regional sample of postpartum women, thereby expanding current understanding of factors associated with fertility attitudes in a European setting characterised by persistently low fertility.

### 4.5. Limitations

Several limitations should be considered when interpreting these findings. First, the cross-sectional design precludes conclusions regarding causality or the temporal direction of the observed associations. Consequently, the mediation analyses should be interpreted as statistical models describing associations among the examined variables rather than demonstrating temporal mediation. Longitudinal studies are needed to clarify how health literacy, psychological distress, and fertility attitudes interact over time.

Second, all variables were assessed using self-report measures, making the results susceptible to recall bias, social desirability bias, and the participants’ current emotional state. Psychological status was assessed using validated questionnaires rather than clinical diagnostic interviews. Furthermore, although the fertility attitudes questionnaire demonstrated good internal consistency, it has not undergone formal international psychometric validation, which may limit comparisons with studies conducted in other populations.

Third, the binary categorisation used in the logistic regression analysis may have resulted in some loss of statistical information compared with modelling the continuous fertility attitude score. Nevertheless, this approach was selected because it addressed a specific demographic and public health research question concerning preferences corresponding to above-replacement fertility and allowed straightforward interpretation of odds ratios.

Fourth, the study included postpartum women from seven coastal counties of Croatia. Consequently, the findings may not be fully generalisable to women from other geographical regions or sociocultural settings. In addition, only women who had recently given birth were included, excluding women postponing parenthood, experiencing infertility, or voluntarily remaining childfree.

An additional limitation relates to the timing of data collection. Participants completed the questionnaires within two to four days after childbirth, a period characterised by substantial physical recovery, hormonal fluctuations, emotional adjustment, and the initiation of breastfeeding. These factors may have temporarily influenced psychological distress, breastfeeding attitudes, and fertility intentions, meaning that the responses may partly reflect experiences specific to the immediate postpartum period rather than more stable long-term attitudes. Nevertheless, collecting data shortly after childbirth reduced the potential influence of subsequent life events and recall bias, thereby providing valuable insight into women’s perceptions during a clinically important period. Future longitudinal studies should determine whether these attitudes remain stable or change throughout the postpartum period.

Finally, the regression model explained only a modest proportion of the variance in preferences corresponding to above-replacement fertility, indicating that important determinants—including relationship characteristics, economic security, value orientations, reproductive history, and broader sociocultural influences—were not captured. Accordingly, the findings should be interpreted as reflecting fertility attitudes during the immediate postpartum period rather than stable attitudes across the entire reproductive life course.

## 5. Conclusions

The findings of this study suggest that fertility attitudes among postpartum women are associated with both psychological well-being and cognitive resources that support informed reproductive decision-making. Higher health literacy remained independently associated with more favourable fertility attitudes and with preferences corresponding to above-replacement fertility, even after adjustment for demographic and psychological characteristics. In addition, older age, lower levels of depressive symptoms, and more positive breastfeeding attitudes were independently associated with preferences corresponding to above-replacement fertility.

Although anxiety and depressive symptoms were associated with fertility attitudes, psychological distress did not explain the association between health literacy and fertility attitudes, indicating that health literacy may represent a distinct cognitive resource in reproductive decision-making. These findings support the importance of considering health literacy alongside psychological well-being when developing strategies to promote reproductive health. Strengthening health literacy while supporting maternal mental health may therefore represent complementary public health approaches to improving reproductive health literacy, informed decision-making, and women’s well-being during the postpartum period.

## Figures and Tables

**Table 1:** Sociodemographic characteristics of the participants (*N* = 1656).

	Category	*N* (%)	Mean (SD)	Median (IQR)
Age			30.6 (5.2)	30 (27–34)
Range (groups)	18–24	196 (12)		
	25–29	452 (27)		
	30–34	581 (35)		
	35–39	424 (26)		
	≥40	3 (0.2)		
Range (total)	18–44			
Marital status	Married	1477 (89)		
	Unmarried	179 (11)		
Educational level	Primary school	298 (17)		
	Secondary school	904 (56)		
	University degree	454 (27)		
Age at first childbirth			29.2 (4.5)	29 (25–31)
Range (groups)	18–24	224 (14)		
	25–29	625 (39)		
	30–34	553 (33)		
	35–39	254 (14)		
	>40	0 (0)		
Economic status	551–1000 €	284 (17)		
	1001–1500 €	967 (60)		
	1501–2000 €	258 (15)		
	>2001 €	147 (8)		
Place of residence	<500	118 (7)		
	500–10,000	823 (50)		
	10,000–100,000	612 (37)		
	>100,000	103 (6)		

**Table 2:** Descriptive results of measurement instruments (*N* = 1656).

Instrument	Mean	SD	Median	Min	Max
NVS-HR	4.7	1.2	5	0	6
STAI-S	41.3	9.8	41	20	76
STAI-T	39.7	8.9	39	20	73
BDI-M	1.3	0.62	1	1	4
IIFAS	67.8	8.4	68	29	85

**Table 3:** Distribution of participants according to depression severity categories (BDI-M) (*N* = 1656).

Depression Category	*N*	%
Minimal depression	1280	77.3
Mild depression	282	17.0
Moderate depression	68	4.1
Severe depression	26	1.6

**Table 4:** Correlation analysis of health literacy, psychological distress, and fertility attitudes.

Factor Associated with	1	2	3	4	5
NVS-HR	1				
STAI-S	−0.21 *	1			
STAI-T	−0.24 *	0.71 *	1		
BDI-M	−0.26 *	0.63 *	0.67 *	1	
Fertility attitudes	0.18 *	−0.22 *	−0.20 *	−0.25 *	1

* *p* < 0.001; Note: Values represent Spearman’s correlation coefficients (or Pearson’s, depending on distribution).

**Table 5:** Factors independently associated with above-replacement fertility preference: multivariable logistic regression analysis (extended with model diagnostics).

Factor Associated with	OR	95% CI	*p*
Age	1.08	1.05–1.12	<0.001
Educational level	0.99	0.94–1.05	0.607
Economic status	1.01	0.97–1.08	0.724
Place of residence	0.97	0.92–1.03	0.294
NVS-HR	1.24	1.12–1.35	<0.001
STAI-S	0.96	0.91–1.01	0.125
STAI-T	0.97	0.92–1.01	0.304
BDI-M	0.78	0.66–0.90	<0.001
IIFAS	1.04	1.01–1.09	0.027
Model: χ^2^ = 47.56; *df* = 9; *p* < 0.001; Nagelkerke *R*^2^ = 0.039			
Hosmer–Lemeshow test: *χ^2^* = 9.68; *df* = 8; *p* = 0.65			
Overall classification accuracy = 66.5% (sensitivity = 42.7%; specificity = 81.6%)			
AUC = 0.63 (95% CI: 0.57–0.68)			

**Table 6:** Direct and indirect effects in the mediation model.

Path	*β*	SE	95% CI	*p*
Direct effects				
NVS-HR → fertility attitudes	0.020	0.007	0.007–0.034	0.004
Indirect effects				
NVS HR → STAI-S → fertility attitudes	−0.000017	0.000082	−0.00018–0.00014	0.836
NVS-HR → STAI-T → fertility attitudes	−0.000133	0.000296	−0.00068–0.00041	0.630
NVS-HR → BDI-M → fertility attitudes	0.000096	0.000298	−0.00049–0.00068	0.746
Total indirect effect	−0.000054	0.000469	−0.00097–0.00087	0.909

Bootstrap samples = 5000. Note: Indirect effects were estimated using the bootstrap method. A mediating effect was considered statistically significant if the 95% bootstrap confidence interval did not include zero. As all confidence intervals for indirect effects include zero, a statistically significant mediating role of anxiety and depression in the relationship between health literacy and fertility attitudes was not confirmed.

## Data Availability

The data presented in this study are available on reasonable request from the corresponding author. The data are not publicly available because they contain sensitive health information collected from postpartum women, and public sharing is restricted by the conditions of the ethical approvals, participant informed consent, and applicable data protection regulations, including the General Data Protection Regulation (GDPR).

## Abbreviations

The following abbreviations are used in this manuscript:

BDI	Beck Depression Inventory
GDPR	General Data Protection Regulation
IIFAS	Iowa Infant Feeding Attitude Scale
NVS	Newest Vital Signs
STAI	State-Trait Anxiety Inventory
STROBE	STrengthening the Reporting of OBservational studies in Epidemiology
